# A New Dressing System Reduces the Number of Dressing Changes in the Primary Total Knee Arthroplasty: A Randomized Controlled Trial

**DOI:** 10.3389/fsurg.2022.800850

**Published:** 2022-05-03

**Authors:** Shilong Su, Juan He, Chenggong Wang, Fawei Gao, Da Zhong, Pengfei Lei

**Affiliations:** ^1^Department of Orthopedics, Xiangya Hospital, Central South University, Changsha, China; ^2^Department of Orthopedics, The First Hospital of Changsha, Changsha, China; ^3^College of Stomatology, Changsha Medical University, Changsha, China; ^4^Hunan Key Laboratary of Aging Biology, Xiangya Hospital, Central South University, Changsha, China; ^5^Department of Orthopedics, The First Affiliated Hospital, College of Medicine, Zhejiang University, Hangzhou, China; ^6^Hunan Engineering Research Center of Biomedical Metal and Ceramic Implants, Xiangya Hospital, Central South University, Changsha, China

**Keywords:** dressing change, total knee arthroplasty, satisfaction, wound, dressing

## Abstract

**Purpose:**

We devised a novel dressing system to accelerate the recovery after total knee arthroplasty (TKA). The purpose of this study was to assess the clinical outcomes and economic expenses of the new dressing system.

**Methods:**

In this randomized and controlled trial, we enrolled 98 patients who underwent the first unilateral TKA between September 2020 and June 2021. The patients were randomly assigned to one of two groups: the intervention (the new dressing system group) or the control (the traditional gauze dressing) group. We gathered and evaluated patient data including age, gender, body mass index, surgical side, number of dressing changes, post-operative hospital stay, dressing-related expense, satisfaction, pain and function scores, wound scores and wound-related complications.

**Results:**

The number of dressing changes and post-operative hospital stay in the intervention group were significantly less than in the control group (*p* = 0.000, *p* = 0.002). Satisfaction in the intervention group was significantly higher than in the control group's (*p* = 0.000). There were no significant differences between the two groups in dressing-related expense, pain and function scores. During the one month follow-up, the intervention group's Stony Brook Scar Evaluation Scale (SBSES) was considerably higher than the control group's (*p* = 0.012).

**Conclusion:**

The new dressing system can reduce the number of dressing changes and post-operative hospital stays while increasing patient satisfaction with no difference in medical costs in TKA. This wound dressing system has potential for application in TKA

**Clinical Trial Registration:**

https://clinicaltrials.gov, identifier ChiCTR2000033814.

## Introduction

Total knee arthroplasty (TKA) is currently one of the most successful procedures. TKA can effectively reduce pain in patients (1, 2). The recovery of patients after TKA is very important, and wound management is crucial in this regard. Any surgery has the risk of surgical wound complications and, in extreme cases, post-operative infection. One of the terrifying consequences of TKA is prosthetic joint infection (PJI), which is one of the main reasons for TKA revision (3). When complications such as PJI occur, recovery time, hospital stay, medical expenses and pain of patients increase, putting a burden on the family and society. prevention of surgical wound complications and PJI is critical throughout the treatment course from admission to rehabilitation.

Many aspects of surgical wound management are involved, including the management of patients' basic diseases (4, 5), proper skin preparation before operation (6), routine antibiotic infection prevention (7), layer-by-layer inverted suture during operation and antibacterial irrigation of operation area (8). Another critical consideration is the selection of wound dressings. Ideal wound dressings features include providing a moist environment to reduce wound dryness, preventing contamination, absorbing wound exudates, stimulating cell growth factors, appropriate mechanical strength and flexibility and biocompatibility/biodegradability (9). All of these can promote wound healing and prevent wound complications, thereby avoiding PJI. Most wound dressings currently employ classic sterile gauze and cotton pads, but gauze and cotton pad dressings are easily contaminated by the external environment, such as wetting, which increases the risk of infection. simultaneously, standard bandages must be changed regularly, increasing the labor of health care professionals, while surgical incisions are repeatedly exposed to pathogens in the surrounding air, increasing the risk of infection (10).

At the moment, smart dressings, especially Aquacel Ag Hydrofiber, can be used in the novel wound care strategy, which is successful in encouraging wound healing and preventing bacterial colonization infection (11). However, the high cost is a major obstacle to its widely used, particularly in developing nations and some low-income patients in rural areas. Therefore, it is critical to develop a low-cost dressing system that can efficiently promote wound healing. Given this clinical demand problem, we devised a new dressing system that is waterproof, breathable, low-cost and capable of reducing the number of dressing changes and we tested its effectiveness and feasibility in prior studies. The combination of IV3000 films and calcium alginate dressing was used to address surgical wounds in TKA patients. It not only utilities use of the benefits of calcium alginate dressing in promoting wound healing and strong ability to absorb wound exudate, but also utilizes characteristics of IV3000 film, such as breathable and waterproof, good skin adhesion and skin-friendly; at the same time, this dressing system is low in cost and suitable for popularizing and using in a large area.

This prospective randomized controlled study (RCT) was conducted to confirm the benefits of this new dressing system over traditional gauze dressings. The number of post-operative dressing changes, post-operative hospitalization days, wound score and complications, functional recovery, medical costs and self-evaluation of satisfaction were all recorded in this study.

## Methods

This RCT was carried out under the principles of the Helsinki Declaration (as revised in 2013) and was authorized by the medical ethics committee of the Xiangya Hospital of Centre South University (No. 202010128). All individual participants in the study gave their informed consent. This RCT was conducted following the CONSORT guidelines and was registered at the Chinese Clinical Trial Registry (ChiCTR2000033814).

### Participants

Patients with clinically and radiographically confirmed osteoarthritis were eligible to participate. The inclusion criteria were as follows: (I) diagnosed with osteoarthritis; (II) first-time TKA; (III) age 18–85 years; and (IV) operation on one side alone. Patients who were unable to complete the regular follow-up, had skin diseases, like psoriasis or eczema, or other serious systemic diseases, had previous open knee surgery on either knee, had previous major trauma to either knee resulting in deformity or scarring or had allergies to skin adhesives were excluded. According to relevant research (10, 12), a minimum of 30 participants was required for each group according to a power analysis (alpha = 0.05, beta = 0.2).

**Figure 1:**
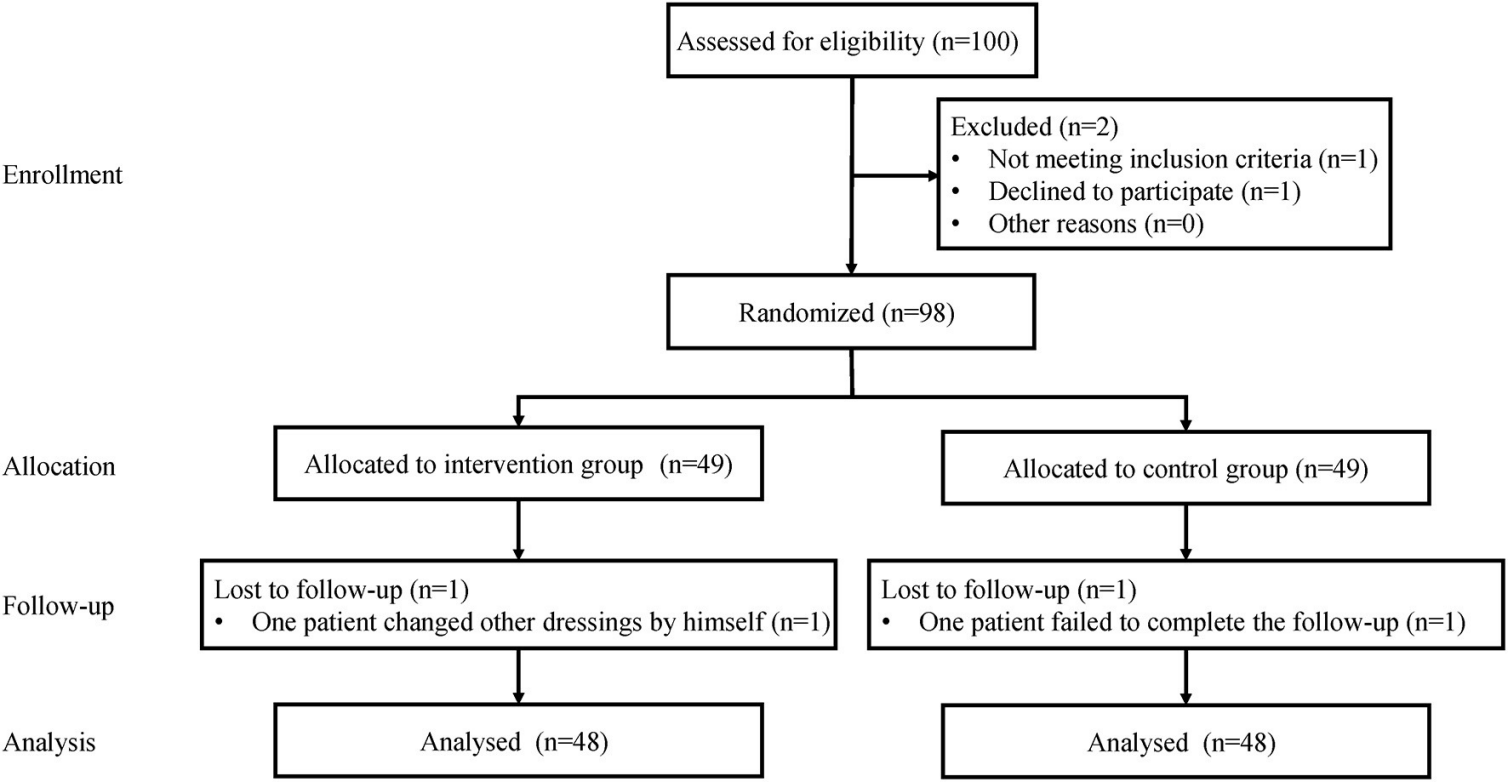
Flow diagram of the allocation of patients to the intervention group (new dressing system group) and the control group (the traditional gauze dressing group).

From September 2020 to June 2021, a total of 100 participants were tested for trial eligibility, with two patients were excluded. A random number generator was used to divide the patients into the intervention group (the new dressing system group) (*n* = 49) (the traditional gauze dressing group) (*n* = 49). The computer-generated randomization technique was carried out using opaque envelopes, which were opened intraoperatively before skin closure of the knee. One patient in the intervention group was removed because he changed his dressings after being discharged. One patient in the control group was excluded after failing to complete the regular one month follow-up was excluded. Finally, 96 patients were included in the analysis (Figure 1).

An expert in joint surgeon performed all TKA operations. A typical midline skin incision with a medial parapatellar capsulotomy was performed on all patients. In all patients, the Attune knee prothesis (DePuy, Warsaw, USA) was applied.

### Application of the Dressing

Cefoxitin, a prophylactic antibiotic, was regularly administered 30 min before surgery. Tranexamic acid (TXA, 1.0 g) was given intravenously twice before exposure and wound closure. Duration the operation, all patients had a standard three-layer continuous suture approach performed in about 45° flexion of the knee. The incision was closed by two fixed residents. The articular capsule was sutured continuously with 2# absorbable knot-free unidirectional barb suture (Quill, Surgical Specialties Corporation, New York, USA), while the subcutaneous tissue was sutured continuously with 0# absorbable knot-free bi-directional barb suture (Quill, Surgical Specialties Corporation, New York, USA). We used 3-0 absorbable knot-free bi-directional barbed sutures in the skin (Quill, Surgical Specialties Corporation, New York, USA).

The new dressing system was used as follows in the intervention group:

1. After suturing the surgical incision, thoroughly deiodinase the skin of 10 cm around the incision with 75% alcohol.
2. Fold the calcium alginate dressing (Algisite M, Smith & Nephew, London, UK) into three layers in the long axis direction and keep it slightly longer than the notch 1 cm.
3. Based on the length of the incision, 3 to 4 pieces of IV3000 film (Smith & Nephew, London, UK) were selected when the knee flexion was about 45°, Following that, glue the middle one first and then the film from the distal end to the proximal end. The two ends of the films were roughly 4 cm longer than the incision and the overlap between the two films was about 1 cm in length. There were no air bubbles between the films and the skin, thus they adhered to the skin.

**Figure 2:**
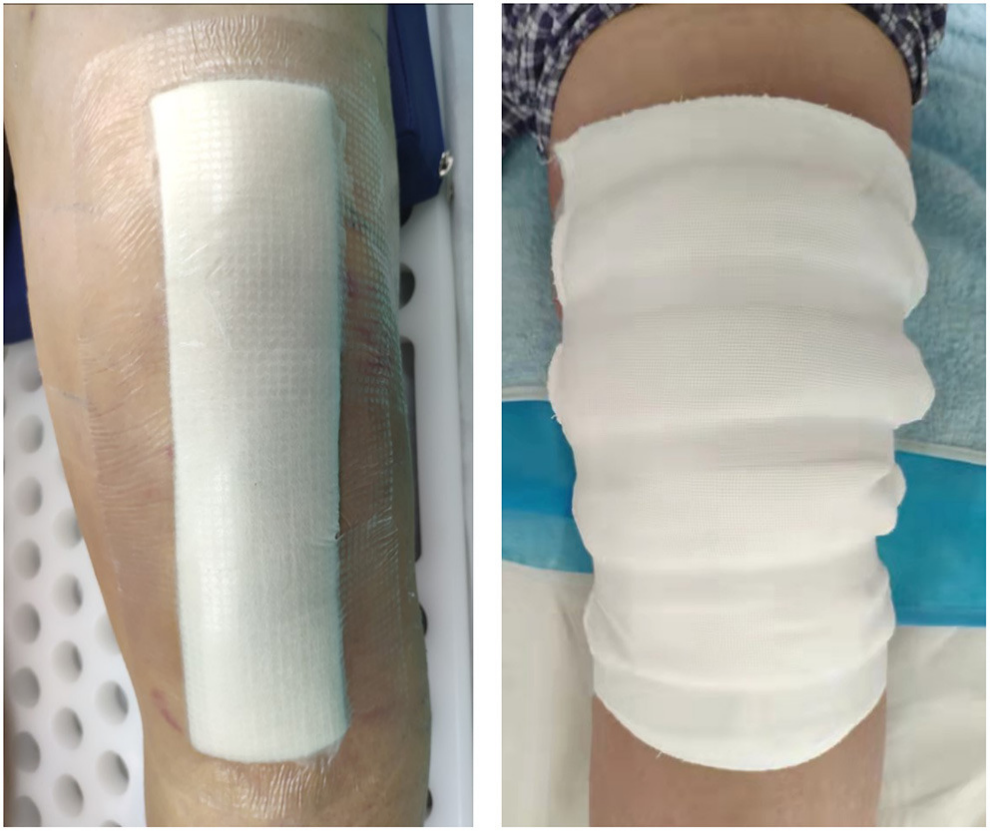
The appearances of wound dressings (left: new dressing system; right: traditional gauze dressing).

In the control group, suturing of the surgical incision, cover the wound with eight layers of aseptic gauze, then one layer of aseptic cotton pad and finally, secure it with plastic tape along the long axis of the wound (Figure 2).

Following the operation, all patients used the same nursing measures. All patients were given antibiotics within 24 h of surgery and 1 g intravenous TXA 3 h later. Patients were given oral thromboembolic prophylaxis in the form of 10-mg rivaroxaban tablets once daily for 14 days beginning 6 to 8 h after surgery. All patients should have their dressings changed when the dressing has been soaked through, the dressing has become loose or fallen off, or the patient felt uncomfortable. Patients using the new dressing system were allowed to bath as usual based on their lifestyle. Patients with traditional gauze dressing can only brush their bodies 2 weeks following the operation, and they are not allowed to take showers or bubble baths to avoid wetting the dressings. All patients were not covered with dressing 2 weeks after the operation. After discharge, the patient was assigned to a chat group and the dressing was photographed and evaluated under the guidance of a fixed medical staff.

### Follow-Up and Data Collection

Each patient's preoperative, intraoperative and post-operative data were collected, including age, gender, body mass index (BMI), surgical site, the times of dressing changes, post-operative hospital stay, the dressing-related cost, satisfaction, pain and functional recovery scores, wound scores and wound-related complications.

### Number of Dressing Changes and Post-operative Hospital Stay

Patients were discharged from the hospital if they met specific criteria, such as the ability to perform independent personal care, walk at least 70 m on crutches, get in and out of bed and get up from chairs and were treated with oral pain relief (13). The post-operative hospital stay was computed as the whole day, whereas the part less than one day was also calculated as one day. After discharge, the patient was assigned to a chat group in which the dressing was photographed and evaluated under the guidance of medical personnel. We kept track of the total number of dressing changes.

### Dressing-Related Cost and Satisfaction

The dressing-related cost was the total cost of the dressing during the treatment cycle. To perform a satisfaction survey, we created a satisfaction survey (Table 1). The satisfaction measure recorded patients' satisfaction with eight categories, including their comfort with dressings, ability to take a bath, pain treatment, physician visits, hospital stay, times of dressing changes, hospitalization costs and overall satisfaction. All measurements were recorded numerically, with a score of 0 to 10, with a maximum score of 80. The patients filled up the data according to their actual circumstances one month after the operation.

**Table 1:** Satisfaction record table.

	**1**	**2**	**3**	**4**	**5**	**6**	**7**	**8**	**9**	**10**
Comfort with dressings										
Ability to take a bath										
Pain treatment										
Physician visits										
Hospital stay										
Times of dressing changes										
Hospitalization costs										
Overall experience										
Total score										

### Pain and Function Score

We use the visual analog scale (VAS) score (14, 15) and The Knee Society Score (KSS) (16, 17) to record the joint pain and function of patients and evaluate the changes of perioperative patients. The evaluation was carried out and data was recorded between 1 week before operation and 1 month after the operation.

### Wound Score and Wound-Related Complications

The Stony Brook Scar Evaluation Scale (SBSES), proposed by Singer et al. (18), is a wound evaluation scale designed to measure the cosmetic effect of a wound, including the width, height, color, remaining suture marks and an overall view of the scar. Each index has a score of 0 or 1 and the total score is calculated, ranging from 0 (worst) to 5 (best). Furthermore, the ASEPSIS score (19) is a widely used wound assessment score that has been suggested for orthopedic infection surveillance (20). This score comprises sections of wound assessment, wound treatment management and infection consequences. We only used the objective wound assessment section of the score in this study (21), because we only wanted to know the clinical appearance of the wound. The SBSES and ASEPSIS scores were recorded one month after the operation. Simultaneously, the wound-related complications of patients were recorded and photographed within one month of operation. Wound-related complications included redness, dehiscence, subcutaneous hematoma, surgical site infection and re-suture for whatever reason.

### Statistical Analysis

The mean ± standard deviation was used to express all quantitative data. Quantitative data was analyzed by the independent samples *t*-test. Qualitative data were analyzed by the chi-square test. Statistical analyses were performed using SPSS 25.0 software (SPSS Inc., Chicago, IL, USA). *P* values < 0.05 were thought to be statistically significant.

## Results

Table 2 shows the demographic features of the remaining 96 patients. There were no significant differences between the two groups in terms of age, gender, BMI, or surgical side. The intervention group's dressing change and post-operative hospital stay were considerably shorter than the control group's (*p* = 0.000, *p* = 0.002). The intervention group's satisfaction was significantly higher than that in the control group (*p* = 0.000) (Table 3). There were no significant differences between the two groups in terms of dressing-related expenses, VAS, or KSS between the two groups.

**Table 2:** Demographic characteristics of the patients.

**Characteristics**	**Intervention group**	**Control group**	***P* value**
Age (years)	64.15 ± 9.06	67.60 ± 9.52	0.071
Gender (male/female)	14/34	8/40	0.145
BMI (kg/m^2^)	26.39 ± 2.90	26.39 ± 2.05	0.995
Surgical side (left/right)	21/27	25/23	0.414

*BMI, body mass index*.

**Table 3:** Comparisons of times of dressing changes, post-operative hospital stay, dressing-related cost, satisfaction, pain and function score between two groups.

**Variables**	**Intervention group**	**Control group**	***P* value**
Dressing change	0.85 ± 0.46	4.71 ± 0.71	0.000
Post-operative hospital stay (days)	4.04 ± 1.24	5.06 ± 1.88	0.002
Dressing-related cost (US dollar)	61.19 ± 15.21	59.37 ± 7.42	0.458
Satisfaction	73.35 ± 3.92	68.56 ± 3.70	0.000
**VAS**			
Pre-op	6.15 ± 1.43	5.94 ± 0.98	0.407
Post-op (1 month)	0.75 ± 0.73	0.92 ± 0.67	0.239
**KSS**			
**Knee**			
Pre-op	57.23 ± 19.22	56.88 ± 19.96	0.930
Post-op (1 month)	87.35 ± 5.05	87.65 ± 4.63	0.769
**Function**			
Pre-op	50.00 ± 25.06	55.29 ± 21.10	0.266
Post-op (1 month)	73.33 ± 8.71	74.17 ± 8.71	0.640

*VAS, visual analog scale; Pre-op, preoperative; Post-op, post-operative; KSS, knee society score*.

**Figure 3:**
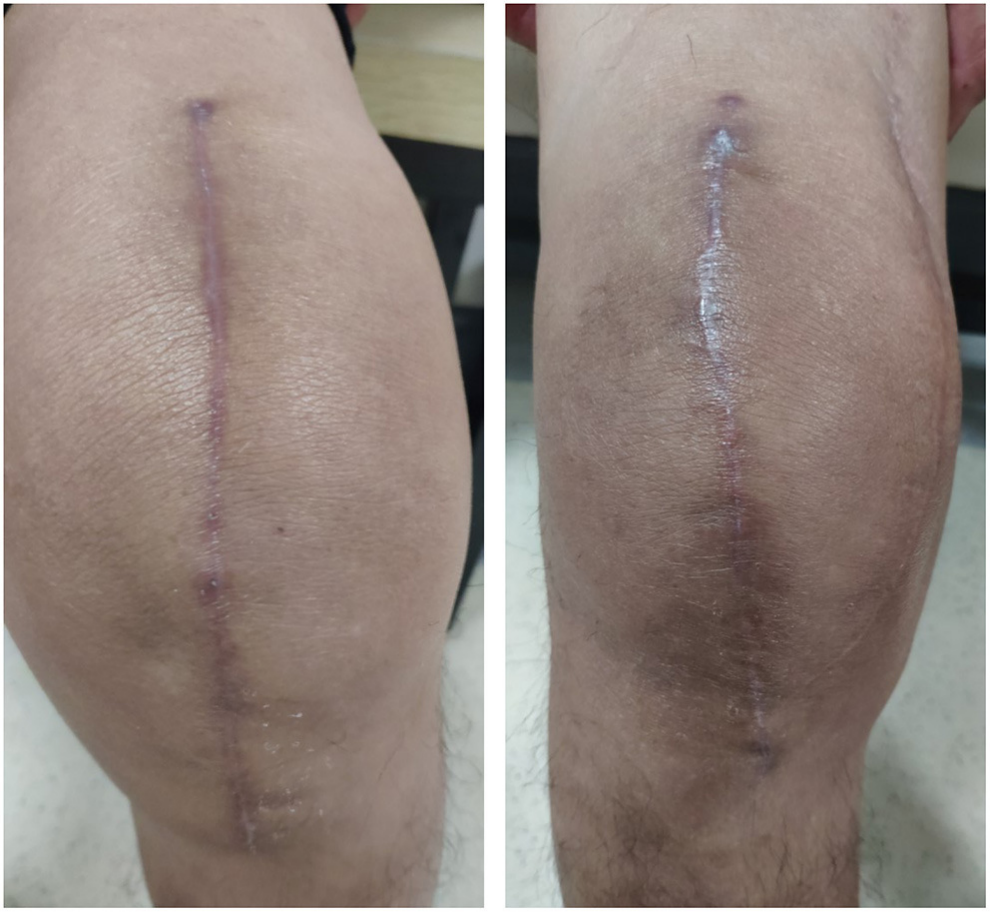
The appearances of knee wound at post-operative one month (right: intervention group; left: control group).

During the one month follow-up, the SBSES in the intervention group was significantly better than that in the control group (*p* = 0.012) in terms of wound score and wound-related complication. There was no significant difference in ASEPSIS between the two groups. However, one patient in the control group had a subcutaneous hematoma on day 2 post-operatively and required pressure dressing and antibiotics for an extended period (Table 4). The wound healed completely before he was discharged. Figure 3 depicts the post-operative one month appearances of the wound in one patient in the intervention group and one from the control group.

**Table 4:** Comparisons of wound score and wound-related complications between two groups.

**Variables**	**Intervention group**	**Control group**	***P* value**
SBSES	4.35 ± 0.73	4.00 ± 0.62	0.012
ASEPSIS	0.00	0.08 ± 0.58	0.322
**Wound-related complications**			
Redness	0	0	
Dehiscence	0	0	
Subcutaneous hematoma	0	1	
Surgical site infection	0	0	
Re-suture	0	0	

*SBSES, stony brook scar evaluation scale*.

## Discussion

The avoidance of surgical wound complications and PJI is critical work for TKA patients. Furthermore, since the notion of enhanced recovery after surgery gained huge attention in joint surgery, to improve wound healing, increase patients' satisfaction and reduce hospitalization days, surgical wound management has become increasingly important. Therefore, after extensive research and development, our team has creatively designed this dressing system by merging IV3000 films and calcium alginate dressing in a specific way. This RCT confirmed that the new dressing system can significantly minimize dressing change time and post-operative hospital stays when compared with traditional gauze dressings. Furthermore, the new dressing system has the potential to greatly improve patients' satisfaction and wound appearance scores.

In general, post-operative patients' wounds are covered with dressings which should be changed regularly until the wound heals. Therefore, selecting the appropriate wound dressing is very important. The optimal dressing for total knee arthroplasty should provide a moist, warm and clean environment to promote wound healing (22); allow the wound to breathe (23); and accommodate motion allowing wide and free range of movements with no risk of friction (24). However, most wound dressings are unable to achieve the aforementioned requirements.

We discovered that our new dressing system meets these conditions through our RCT. First, the new dressing system is permeable enough to control a wet environment while absorbing adequate exudate. And it can significantly reduce the times of dressing changes and post-operative hospital stay compared with traditional gauze dressings. On the one hand, it reduces the risk of wound infection and pain caused by frequent dressing changes, while on the other hand, it avoids patients' poor lack of wound management awareness of the risk of irregular dressing changes. simultaneously, the decrease in the frequency of dressing changes and hospitalization days has also reduced the cost of medical care. We also observed that the patients had a higher score of wound healing appearance. Second, the new dressing system can significantly increase patients' satisfaction. This is because it requires fewer dressing changes, which lowers the pain of dressing changes, and it has good elasticity and skin-friendliness, allowing it to fully satisfy the needs of the knee joint post-operative exercise. Furthermore, the new dressing system is waterproof and can accommodate patients' usual washing habits, which is critical to improving patients' quality of life and lowering the risk of infection after surgery. The selection of post-operative dressing should be based on a compromise between evidence-based and patient-focused considerations. After all, it is the patient who is the true user and experiencer. The relevance of satisfaction record tables for evaluating patient comfort cannot be overstated (25). At present, there are few studies in this area (26).

The cost of a single change of dressing in the new dressing system is much more than that of traditional gauze dressings, which we are concerned will raise the economic cost of dressings. However, there is no increase in the economic cost of patients for dressing-related charges during the treatment cycles. This is different from some smart dressings. At present, the cost of most smart dressings is high and it is difficult to popularize and use them in a large area (11, 27). We used the scoring method to access the patient's post-operative knee pain and function compared the two groups and discovered no statistical difference. We feel that the current scoring method is primarily intended at evaluating joint function recovery after surgery is mostly related to the surgeon's technical experience, the type of prosthesis chosen and the situation of post-operative rehabilitation exercise. The management of wound dressings only accounts for a small part of them and the positive role it can play in the whole perioperative period is limited. The scoring system, thus reflects that there is no significant statistical difference in the score between the two groups. Kong et al. (12) and Langlois et al. (28) conducted the same evaluation and found the same results.

Of course, this study also has limitations, first of all, this study is a single-center study, the sample size is small, the follow-up time is limited to 1 month following the operation. A larger sample size and longer follow-up time are needed to provide more abundant data. In terms of prospective controlled clinical trials and wound healing periods, it has produced sufficient knowledge and data. Second, we only compared patients who received unilateral TKA and could not eliminate their deviations, such as BMI, blood clotting disorders and personal wound healing ability. Therefore, it is necessary to study patients who receive bilateral TKA. Third, due to the uniqueness of wound dressing, the blind method cannot be used and there may be a tendency deviation in the subjective score section. Fourth, because the cost of dressings varies by region and institution, the cost evaluation in this study may not apply to other institutions.

## Conclusion

The new dressing system can reduce the times of dressing changes and post-operative hospital stay while increasing patients' satisfaction with no difference in medical costs in TKA. In TKA the new dressing system is a promising wound dressing.

## Data Availability Statement

The original contributions presented in the study are included in the article/supplementary materials, further inquiries can be directed to the corresponding authors.

## Ethics Statement

Written informed consent was obtained from the individual(s) for the publication of any potentially identifiable images or data included in this article.

## Author Contributions

DZ and PL conceived the original ideas of this manuscript. SS, JH, and FG executed the follow-up examination and materials collection. DZ, CW, and PL read the examination results, participated in the surgical, and medical treatment. SS prepared the figures. PL and SS prepared the manuscript. All authors contributed to the article and approved the submitted version.

## Funding

This study was supported by the National Natural Sciences Foundation of China (Grant Nos. 81974360 and 81902308) and the Innovation Foundation of National Orthopedics and Sports Rehabilitation Clinical Medicine Research Center (Grant No. 2021-NCRC-CXJJ-PY-37).

## Conflict of Interest

The authors declare that the research was conducted in the absence of any commercial or financial relationships that could be construed as a potential conflict of interest.

## Publisher's Note

All claims expressed in this article are solely those of the authors and do not necessarily represent those of their affiliated organizations, or those of the publisher, the editors and the reviewers. Any product that may be evaluated in this article, or claim that may be made by its manufacturer, is not guaranteed or endorsed by the publisher.
